# A supramodal role of the basal ganglia in memory and motor inhibition: Meta-analytic evidence

**DOI:** 10.1016/j.neuropsychologia.2017.11.033

**Published:** 2018-01-08

**Authors:** Yuhua Guo, Taylor W. Schmitz, Marieke Mur, Catarina S. Ferreira, Michael C. Anderson

**Affiliations:** a MRC Cognition and Brain Sciences Unit, 15 Chaucer Road, Cambridge CB2 7EF, UK; b University of Cambridge, The Old Schools, Trinity Ln, Cambridge CB2 1TN, UK; c University of Birmingham, Edgbaston, West Midlands, Birmingham B15 2TT, UK

**Keywords:** AAL, Anatomical Automatic Labelling, ADHD, attention deficit hyperactivity disorder, ALE, Activation Likelihood Estimation, ATAG, Atlasing of the Basal Ganglia, CMA, Centre for Morphometric Analysis, DLPFC, dorsolateral prefrontal cortex, FEF, frontal eye field, fMRI, functional magnetic resonance imaging, FWHM, full width at half maximum, GPe, external globus pallidus, MNI, Montreal Neurological Institute, M1, primary motor cortex, rIFG, right inferior frontal gyrus, STN, subthalamic nucleus, SN, substantia nigra, SNc, substantia nigra pars compacta, SNr, substantia nigra pars reticulata, VMPFC, ventromedial prefrontal cortex, Basal ganglia, Cognitive control, Meta-analysis, Memory inhibition, Motor inhibition

## Abstract

The ability to stop actions and thoughts is essential for goal-directed behaviour. Neuroimaging research has revealed that stopping actions and thoughts engage similar cortical mechanisms, including the ventro- and dorso-lateral prefrontal cortex. However, whether and how these abilities require similar subcortical mechanisms remains unexplored. Specifically of interest are the basal ganglia, subcortical structures long-known for their motor functions, but less so for their role in cognition. To investigate the potential common mechanisms in the basal ganglia underlying action and thought stopping, we conducted meta-analyses using fMRI data from the Go/No-Go, Stop-signal, and Think/No-Think tasks. All three tasks require active stopping of prepotent actions or thoughts. To localise basal ganglia activations, we performed high-resolution manual segmentations of striatal subregions. We found that all three tasks recovered clusters in the basal ganglia, although the specific localisation of these clusters differed. Although the Go/No-Go and Stop-signal tasks are often interchangeably used for measuring action stopping, their cluster locations in the basal ganglia did not significantly overlap. These different localised clusters suggest that the Go/No-Go and Stop-signal tasks may recruit distinct basal ganglia stopping processes, and therefore should not be treated equivalently. More importantly, the basal ganglia cluster recovered from the Think/No-Think task largely co-localised with that from the Stop-signal task, but not the Go/No-Go task, possibly indicating that the Think/No-Think and Stop-signal tasks share a common striatal circuitry involved in the cancellation of unwanted thoughts and actions. The greater similarity of the Think/No-Think task to the Stop-Signal rather than Go/No-Go task also was echoed at the cortical level, which revealed highly overlapping and largely right lateralized set of regions including the anterior DLPFC, VLPFC, Pre-SMA and ACC. Overall, we provide novel evidence suggesting not only that the basal ganglia are critical for thought stopping, but also that they are involved in specific stopping subprocesses that can be engaged by tasks in different domains. These findings raise the possibility that the basal ganglia may be part of a supramodal network responsible for stopping unwanted processes more broadly.

## Highlights

• Stopping actions and thoughts both consistently activate the basal ganglia.
• Action prevention and action cancellation engage distinct basal ganglia processes.
• Thought stopping co-localises with action cancellation, but not prevention.
• Basal ganglia may support a supramodal process cancellation mechanism.

## 1 Introduction

Being able to stop actions and thoughts is fundamental to goal-directed behaviour. Much research has sought to understand how people stop prepotent responses when needed, a process known as inhibitory control. Although research on inhibitory control has often focused on stopping motor actions, there has also been significant interest in how people stop higher-level cognitive processes, such as memory retrieval. Recent evidence from neuroimaging studies suggests that inhibiting motor actions and memory retrieval may engage similar cortical mechanisms, and that a supramodal inhibition mechanism may be supported in part by the right dorsolateral and ventrolateral prefrontal cortices (DLPFC, VLPFC; Depue et al., 2015). Although there have been previous meta-analyses on the common activations across motor inhibition tasks (Cai et al., 2014, Rae et al., 2014, Swick et al., 2011), no studies have examined whether memory inhibition consistently activates similar regions. In addition, previous studies usually focused analyses on the cortical level. In the case of motor inhibition, subcortical mechanisms are known to contribute significantly, particularly the basal ganglia (Aron et al., 2014; Rae et al., 2014). Moreover, whether and how the basal ganglia are engaged in memory inhibition remains unknown. Here we conducted a quantitative meta-analysis to examine the basal ganglia contribution to both memory and motor inhibition, with a particular emphasis on how people stop retrieval. Moreover, we consider whether scrutinising the specific localisation of domain-specific activations in the basal ganglia might contribute to our understanding of the roles of the basal ganglia in inhibiting memories and actions.

The basal ganglia are a group of subcortical nuclei with a well-established role in motor inhibition (Aron et al., 2007, Graybiel, 2005, Kandel et al., 2012). Studies from the animal literature have identified three coordinating pathways in the basal ganglia that contribute to the different processes in motor control: the hyperdirect, direct, and indirect pathways (Alexander and Crutcher, 1990; Nambu et al., 2002). The hyperdirect pathway has two primary roles (Takada et al., 2013): first, it globally inhibits all motor responses to prevent unnecessary movements from taking place prior to movement onset and second, it engages an early selection process that implicitly determines the ideal goal-directed motor response. Following the hyperdirect pathway, the direct pathway initiates the selected motor response. Finally, the indirect pathway terminates the selected motor response either when it is achieved or when it needs to be cancelled (Freeze et al., 2013). Cancellation of an ongoing motor response may be required when the course of the movement needs to be adjusted due to sensory feedback (Fourneret and Jeannerod, 1998), or when the movement needs to be completely revoked due to altered task goals, such as when a stop signal is delivered in a stop-signal task.

The foregoing observations support the idea that the basal ganglia are indispensable for motor inhibition, and that the hyperdirect and indirect pathways are particularly relevant. Whereas the indirect pathway involves cortical input to the striatum, which relays signals to the external globus pallidus (GPe), the subthalamic nuclei (STN), and output via the internal globus pallidus (GPi) and substantia nigra pars reticulata (SNr), the hyperdirect pathway instead involves direct cortical input to the STN and output through the GPi/SNr. Critically, human neuroimaging studies of motor inhibition have provided converging evidence for the importance of basal ganglia structures, such as the STN (Aron and Poldrack, 2006). However, no effort has yet been made to characterise the specific regions within the basal ganglia that are consistently recruited by motor inhibition in humans. In this paper, we address this objective with a meta-analytic approach, which provides quantitative inference of the spatial reliability of activations reported across multiple neuroimaging experiments. To localise the clusters of activation observed at the meta-analytic level, we performed high-resolution manual segmentation of basal ganglia sub-regions.

Although basal ganglia mechanisms are clearly essential for motor inhibition, it is unclear to what extent they are dedicated to motor inhibition per se. It is possible, for example, that some functions performed by the basal ganglia during stopping may apply more broadly to higher-order cognitive processes (Alexander et al., 1986, Schroll and Hamker, 2013). Both patient and neuroimaging studies suggest that at least some high-level cognitive functions are supported by the basal ganglia. For example, patients with basal ganglia impairments such as Parkinson's disease develop deficits in cognitive functions including executive functions and working memory, on top of their motor deficits (Robbins and Cools, 2014). In addition, children with attention deficit hyperactivity disorder (ADHD) consistently show dysfunctions in the frontostriatal network, usually associated with the cognitive control of attention and executive functions (Booth et al., 2005, Durston et al., 2003). Neuroimaging studies with healthy participants also suggest that the basal ganglia are similarly involved in cognitive functions such as working memory gating (Scimeca and Badre, 2012). These findings support the possibility that the basal ganglia are involved in control in general, and thus may play a supramodal role in the inhibitory control of both motor and cognitive processes. Alternatively, the basal ganglia could support distinct, modality-dependent stopping mechanisms that stop motor and cognitive processes (Pauli et al., 2016). The current meta-analysis thus examines the existing literature and delineates whether memory and motor inhibition yield shared or anatomically dissociated subregions of the basal ganglia.

In addition to localising consistent basal ganglia activations, the current meta-analysis may also help to deduce cortical regions contributing to basal ganglia involvement during inhibitory control. Different subregions in the basal ganglia receive largely topographical projections from cortical areas with different functional roles (Haber, 2003, Lawrence et al., 1998, Pauli et al., 2016, Seger, 2013). For example, Haber et al. (2006) traced corticostriatal projections in macaque monkeys from regions associated with different functions, such as the DLPFC (executive functions) and the ventromedial prefrontal cortex (VMPFC; reward processing). Haber et al. found that DLPFC projects to dorsal striatum, including the caudate nucleus, spanning across the internal capsule to medial putamen. The caudate targets were largely in the caudate head, ranging into the caudate body, whereas the putamen targets were almost exclusively rostral to the anterior commissure. In contrast, Haber found that the VMPFC projects primarily to ventral striatum. Similar pathways may exist in humans (Haber and Knutson, 2010). These observations may be important because of evidence that DLPFC plays an essential role in supramodal inhibitory control over multiple task domains, including cognition, emotion, and motor action (Depue et al., 2015). Hence the DLPFC-striatum pathway demonstrated by Haber et al. (2006) could be a candidate through which both memory and motor inhibition are achieved. It is therefore necessary to segment striatal subregions to localise meta-analytic activation clusters in the basal ganglia, including the caudate head, body, and tail sections, and the putamen. This localisation could help us to ascertain whether both memory and motor inhibition engage a DLPFC-striatum pathway and, if so, whether striatal activations are similarly localised.

**Fig. 1.1:**
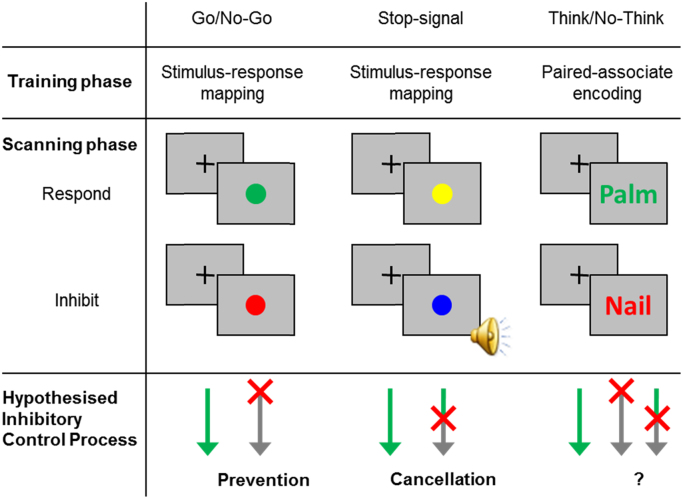
**Typical Go/No-Go, Stop-signal, and Think/No-Think Paradigms and the Hypothesised Inhibitory Control Processes**. In the hypothesised inhibitory control process panel, the arrows denote the time-flow within a single trial. The colour green represents the respond processes, the red “X” represents when inhibitory control is putatively engaged in the trial, and the grey represents the inhibited processes. On a Go or Think trial, participants would carry out the motor response or memory retrieval, respectively. On an inhibit trial, if prevention processes are engaged, inhibitory control should be effective from the very beginning of the trial, before the corresponding response is even started. If cancellation processes are engaged, inhibitory control would be recruited only to terminate an initiated response. In the lower right, the uncertain positioning of the “X” indicates that we do not know whether prevention or cancellation may be more important for the Think/No-Think task.

To address the preceding objectives, we conducted quantitative meta-analyses of brain activations from functional magnetic resonance imaging (fMRI) studies of motor and memory inhibition, including the Go/No-Go, Stop-signal, and Think/No-Think tasks (Fig. 1.1). In a typical Go/No-Go task, participants are presented with visual stimuli, such as red and green circles. When they see some stimuli (e.g., green circles), they need to respond with a motor action, such as pressing a button (known as Go trials). In contrast, upon encountering other stimuli (e.g., red circles), they need to refrain from making any motor responses at all (No-Go trials). The procedure ensures that go trials are much more frequent than No-Go trials so that participants get into the habit of making the button press response. A typical Stop-signal task is similar to the Go/No-Go task. Participants also need to view visually presented stimuli and either carry out a motor response on a Go trial or stop a motor response on a Stop trial. However, in the Stop-signal task, all stimuli represent Go trials, except when an independent stop signal (e.g., an auditory tone) is presented sometime after stimulus onset, signalling the participant to stop. Taking the coloured circles example, participants need to respond to both the blue and green circles, except when a ‘beep’ tone is played shortly after either a blue or a green circle appears, indicating that they need to cancel the motor response. The Go/No-Go and Stop-signal tasks are often treated equivalently in the literature (Nigg, 2000, Zheng et al., 2008), although this might not be the case for reasons we will discuss shortly. Finally, the Think/No-Think task requires participants to stop a cognitive operation, namely, memory retrieval. In a typical Think/No-Think task, participants first learn cue-target associations, such as word pairs (Anderson and Green, 2001), word-object pairs (Gagnepain et al., 2014), or object-scene pairs (Catarino et al., 2015). In the subsequent Think/No-Think phase, each trial presents one cue from one of the pairs. Upon seeing the cues, participants need to either recall the corresponding target to the presented cue if it appears in green (Think trial) or to refrain from recalling the target if the cue appears in red (No-Think trial). A surprise cued-recall test is given at the end of the experiment to measure how recall performance was influenced by retrieving the associated items (Think condition) or by inhibiting their retrieval (No-Think). To measure these effects, recall of Think and No-Think pairs is compared to recall for baseline pairs that were also learned initially, but that were omitted from the Think/No-Think phase.

All three of the foregoing tasks share the feature of having to stop an active process in either the memory or the motor domain. These tasks thus provide the opportunity to investigate the possibility that a supramodal inhibitory control mechanism contributes to stopping processes in general. If so, the meta-analytic clustering observed in each task may co-localise in the basal ganglia. However, each task may also engage different sub-processes through which stopping is achieved. For example, for motor inhibition, Schachar et al. (2007) defined two different forms of stopping required by the Go/No-Go and Stop-signal tasks. On one hand, the Go/No-Go task potentially allows participants to prevent a motor response before it is even initiated: upon recognising a No-Go stimulus, participants could decide not to prepare for a movement, and hence prevent any motor response entirely. On the other hand, the Stop-signal task presents the stop-signal after the cue stimulus appears. Because of this delay in stop-signal onset, participants have likely initiated preparation or execution of the motor response, requiring them to cancel the action. It is unclear whether these different demands (prevention versus cancellation) engage distinct sub-processes that are implemented by different mechanisms within the basal ganglia, such as the hyperdirect pathway for action prevention, and the indirect pathway for action cancellation. For example, whereas Eagle et al. (2008) suggested that the Go/No-Go and Stop-signal tasks have a similar anatomical basis but distinct neuropharmacological underpinnings, Dalley et al. (2011) argued that the tasks engage different brain regions due to the different sub-processes. Specifically, according to Dalley et al. stop-signal tasks primarily activate the right inferior frontal gyrus (rIFG), whereas Go/No-Go tasks activate the left IFG. Within the basal ganglia, the specific regions involved in different tasks or sub-processes remain unresolved, although recent studies have emphasised the role of the STN in the stop-signal task (Aron et al., 2014). The current meta-analysis would therefore be invaluable for examining both whether and how the basal ganglia contribute to different motor inhibition sub-processes. If domain- or sub-process-specific mechanisms are engaged during action prevention and cancellation, the meta-analytic clusters observed in each task may rely on anatomically distinct subregions of the basal ganglia. One possibility, for example, is that Go/No-Go activations may be left lateralised, whereas Stop-signal activations could be right-lateralised, mirroring Dalley's lateralisation proposal for prefrontal cortical involvement in these tasks.

Unlike motor inhibition, there have been no formal studies of whether the basal ganglia consistently contribute to memory inhibition. We therefore used this meta-analysis to examine basal ganglia activations during the Think/No-Think task, and to compare any findings to activations observed during the Go/No-Go and Stop-signal tasks. If memory inhibition engaged the basal ganglia, it is unclear which motor inhibition task it might most resemble (see Fig. 1.1, lower right panel). On one hand, the Think/No-Think task is procedurally similar to the Go/No-Go task because participants are cued to either engage memory retrieval or to stop it, depending on the stimulus presented. On the other hand, the sub-processes required by the Think/No-Think task may instead resemble those engaged by the Stop-signal task. Although the colour of the stimulus instructs participants (on No-Think trials) to prevent the target from being recalled (potentially before the retrieval process gets underway), this attempt at retrieval prevention often fails initially; thus the target memory often intrudes into awareness (Levy and Anderson, 2012). It is possible that overcoming intrusions requires the cancellation of an ongoing retrieval process, making the No-Think task potentially similar to the Stop-signal task. To examine this possibility, Levy and Anderson (2012) asked participants to report whether the target had intruded after each No-Think trial, and found that, indeed, overcoming an intrusion induced larger hippocampal down-regulation than did preventing an intrusion. In addition, Benoit et al. (2014) found that overcoming intrusions triggered greater inhibitory modulation of the hippocampus by the DLPFC. Moreover, similar to cancelling motor actions, inhibiting memory retrieval in the Think/No-Think task primarily recruits right lateral prefrontal cortex (Benoit et al., 2014). Taken together, these findings suggest that the Think/No-Think task engages stopping processes that may be better described as cancellation than prevention. To examine whether memory inhibition requires a sub-process more similar to prevention or cancellation, this meta-analysis will assess whether reported activations across 16 fMRI studies reliably cluster in the basal ganglia, and moreover, how the spatial localisation of any such clusters relate to those observed in the Go/No-Go and Stop-signal tasks. Finally, if basal ganglia clusters are reliable across all three tasks, we will also examine their spatial overlap with conjunction analyses.

To examine the inhibitory control mechanisms in the basal ganglia in memory and motor inhibition, we compared meta-analytic activations both qualitatively by localising the clusters to specific basal ganglia structures, and quantitatively by computing conjunction and contrast maps between tasks. This coordinate-based meta-analysis approach is convenient for illustrating common activations across studies and task modalities. Nevertheless, this analysis method is, by design, more sensitive to spatially clustered activations than it is to activations that are more dispersed. Thus, if a given task does not yield significant activations in a region, by this analysis, it could either be because few activations occurred in that structure across studies, or instead because the activations were spatially dispersed. To distinguish these possibilities, we therefore subsequently visualised the activation foci in the basal ganglia, and computed descriptive summary statistics from the visualised data. These descriptive results give an impression of the dispersion of basal ganglia activations in each of the Go/No-Go, Stop-signal, and Think/No-Think tasks, and with that, provide clues as to whether an absence of significant activation clusters from the meta-analysis of a given task is driven by the absence of activity in the basal ganglia, or instead by a lack of clustered activity.^1^

To characterise the specific localisation of basal ganglia activations, we manually segmented the caudate head, body, tail, and putamen subregions of the striatum, as existing atlases either do not have these subregions available, or have imprecise segmentations. For the other fine nuclei in the basal ganglia, we used an existing ultra-high resolution basal ganglia atlas (Keuken et al., 2014). As suggested by previous findings, we hypothesised that if a supramodal inhibition mechanism existed in the basal ganglia, the task-induced clusters should overlap extensively with each other, possibly in the caudate head and anterior putamen that receive projections from the DLPFC (Haber, 2006). However, if inhibitory control is achieved in a domain-specific or process-specific fashion, the basal ganglia clusters may be distinct across tasks. Specifically, if basal ganglia involvement is domain-specific, there should be co-localised clusters between the motor inhibition tasks (i.e. Go/No-Go and Stop-signal), which differ spatially from clusters observed in the memory inhibition task (i.e. Think/No-Think). However, if basal ganglia involvement is process-specific, there should be co-localised clusters in tasks requiring cancellation of ongoing cognitive or motor operations (i.e. Think/No-Think and Stop-signal), which differ spatially from clusters observed in the task that primarily engages prevention of motor responses (i.e. Go/No-Go). If this pattern is observed, it would raise the possibility of a supramodal basal ganglia contribution to the cancellation of both actions and thoughts. Finally, it was also of interest to examine whether the STN is engaged by the memory and motor inhibition tasks, or whether STN activation is specific to certain tasks or processes.

## 2 Material and methods

### 2.1 Selection criteria and the meta-analytic approach

Studies using the Go/No-Go and Stop-signal tasks were selected for the motor inhibition meta-analyses, whereas studies using the Think/No-Think task were selected for the memory inhibition meta-analysis. For the motor inhibition meta-analyses, we first identified studies from existing ALE meta-analyses (Cai et al., 2014, Rae et al., 2014, Swick et al., 2011). We then searched for additional studies that were published after the above meta-analyses through Google Scholar, using the key words “Stop-signal task” and “Go/No-Go task”, respectively. For the memory inhibition analysis, we included all published Think/No-Think studies, and two additional studies from the laboratory of the last author that are being prepared for publication. These searches resulted in 46 Stop-signal studies, 38 Go/No-Go studies, and 16 Think/No-Think studies. We further screened these studies according to the following criteria:

1. Only fMRI studies reporting results from whole brain analyses in a standardised coordinate space (MNI or Talairach) were included;
2. Only data from healthy adults were included;
3. Only Stop-signal and Go/No-Go tasks where participants responded by hand were included;
4. Only contrasts concerning differences between inhibition and an active condition were included, i.e. No-Think>Think, Stop>Go, and No-Go>Go. We requested the relevant data from each author if they were not already reported in the original article.

According to these criteria, 16 Think/No-Think, 39 Stop-signal, and 30 Go/No-Go studies were identified (Supplement) and included in the meta-analyses. The meta-analyses were conducted using Activation Likelihood Estimation with GingerALE v2.3.6 (Eickhoff et al., 2009, Eickhoff et al., 2012, Eickhoff et al., 2017, Turkeltaub et al., 2012). The following default settings were applied: less conservative mask size; non-additive ALE method (Turkeltaub et al., 2012); no additional FWHM; cluster analysis peaks at all extrema. Where applicable, coordinates reported in Talairach space in the original studies were transformed into MNI space using the icbm2tal transform in GingerALE (Laird et al., 2010, Lancaster et al., 2007) prior to the analyses.

The first step of the meta-analytic approach is to examine the spatial convergence across different studies within each task domain. To do this, three separate meta-analyses were conducted for the Think/No-Think, Stop-signal, and Go/No-Go tasks using cluster-level inference (*p*<.05, cluster-forming threshold uncorrected *p*<.001, threshold permutations=1000). Secondly, to examine the spatial convergence and divergence between different task domains, contrast analyses (Eickhoff et al., 2011) were conducted between each pair of the Think/No-Think, Stop-signal and Go/No-Go Tasks (i.e., Think/No-Think & Stop-signal; Think/No-Think & Go/No-Go; Stop-signal & Go/No-Go). For analysing each pair of the tasks, the thresholded activation maps from the individual analyses, as well as the pooled results from both tasks were used as inputs. The outputs were conjunction and contrast maps between the conditions. The same GingerALE settings were applied to the contrast analyses (less conservative mask size; non-additive ALE method; no additional FWHM; cluster analysis peaks at all extrema.). The results were thresholded to voxel-wise uncorrected *p*<.001, with the *p*-value permutations of 10,000 iterations, and the minimum cluster volume of 200 mm^3^. We present results from all conjunction and contrast analyses between each pair of the tasks.

The ALE analysis is a whole-brain analysis, and we report both cortical and subcortical findings. However, given the conceptual focus on the role of the basal ganglia in memory and motor inhibition, we accordingly focus our consideration of our results on the basal ganglia. In addition to the meta-analytic results, to give an impression of the activation peaks from the included studies, we display their peak coordinates on 3D renders of the basal ganglia. By definition, the ALE analyses are sensitive to common clusters of activation across studies. Activation peaks that are more spatially dispersed, either because a particular cognitive function is not associated with localised activation, or because the included studies are few in number, might therefore not be detected as common across studies. We therefore also counted the number of coordinates that were located in the basal ganglia in the left and right hemispheres. Together, the peak coordinates and their counts serve to provide a comprehensive descriptive picture of the meta-analytic data that the ALE results are based on. We report this information at the end of the results section.

### 2.2 Basal ganglia ROI definition

**Fig. 2.1:**
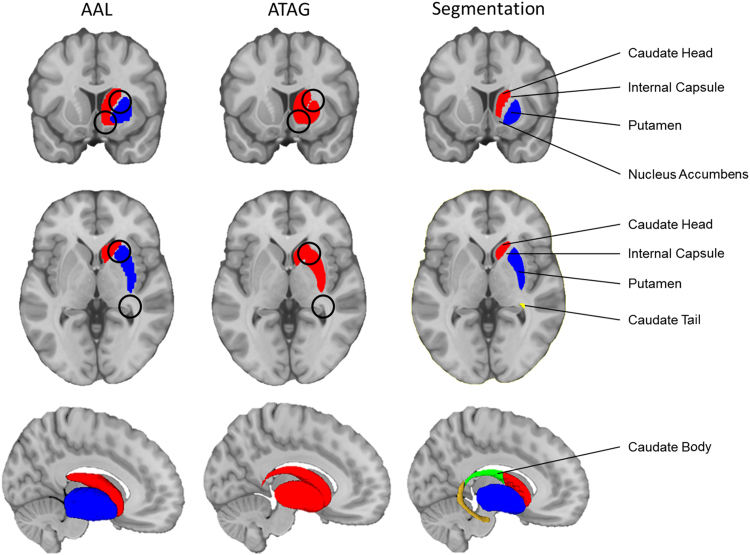
**Segmentation of the striatal subregions**. The three columns compare the AAL and ATAG atlases with our manual segmentation. The top row shows the coronal section, the middle row shows the axial section, and the bottom row shows the 3D rending of the structures in the sagittal plane. The relevant structures are labelled, and the differences are marked with black circles. Anatomical underlay and subcortical renders are displayed in MNI space.

To examine how clusters recovered from the meta-analyses of the memory and motor inhibition tasks related to the subregional anatomy of the basal ganglia, we projected the clusters onto 3D renderings of the subregions. These regions of interest (ROIs) in the basal ganglia were defined with both manual segmentation and an existing atlas (Atlasing of the Basal Ganglia; ATAG; Keuken et al., 2014). Although the ATAG atlas took averages of structural images from ultra-high resolution 7 T MRI and thus provides very fine details of basal ganglia structures, it only treated the striatum as one single structure. No other existing atlases provided high-resolution parcellations of the relevant striatal subregions. We therefore performed manual segmentation of the striatal subregions, including bilateral caudate head, body, tail, and putamen, according to established anatomy and segmentation protocols (Eliez et al., 2002; Levitt et al., 2002; Nolte, 2013); segmentation guidelines provided by the Centre for Morphometric Analysis (CMA; http://www.cma.mgh.harvard.edu/manuals/segmentation/). The segmentations were performed using ITK-SNAP v3.2 (Yushkevich et al., 2006; www.itksnap.org) from the high-resolution ICBM 2009b template structural image (.5 mm isotropic; Fonov et al., 2009, Fonov et al., 2011). Together, these segmentations of the human caudate and putamen improve upon the anatomical precision of several widely used atlases, such as Anatomical Automatic Labelling in SPM (AAL; Tzourio-Mazoyer et al., 2002) and Atlasing of the Basal Ganglia (ATAG). Fig. 2.1 compares our segmentation with these atlases. The resulting subcortical clusters are projected onto the 3D rendering of the segmented structures using Mango v4.0 (Lancaster & Martinez; http://ric.uthscsa.edu/mango/).

#### 2.2.1 Segmentation protocols for the striatal subregions

##### 2.2.1.1 Caudate head

The head of the caudate was segmented through the coronal plane, starting from the slice where it first appears in between the lateral boundaries of the lateral ventricle and the internal capsule, ending at the posterior edge of the anterior commissure, cutting in the middle of the interventricular foramen of Monroe across the frontoparietal axis (Eliez et al., 2002, Levitt et al., 2002, Nolte, 2013). Care was taken not to include the sheet of meninges between the lateral ventricle and the caudate.

The nucleus accumbens was excluded from the caudate head following guidelines provided by the Centre for Morphometric Analysis (CMA) for creating the Harvard-Oxford Subcortical Atlas (http://www.cma.mgh.harvard.edu/manuals/segmentation/). See Fig. 2.1 for an example of this parcellation error in the AAL.

##### 2.2.1.2 Caudate body

The body of the caudate was segmented through the coronal plane, starting from the posterior edge of the anterior commissure until the slice where the cerebral aqueduct enlarges to form the opening of the fourth ventricle (Eliez et al., 2002, Nolte, 2013). The dorsal and ventral boundaries of the caudate body were refined in the sagittal plane, following the lateral ventricle and the internal capsule.

##### 2.2.1.3 Caudate tail

The tail of the caudate started from the coronal slice containing the opening of the fourth ventricle, and was followed until it curved around the thalamus in the sagittal plane. The rest of the tail was traced cross-referencing the coronal, sagittal, and axial planes until it reaches the amygdala.

##### 2.2.1.4 Putamen

The putamen was traced through the coronal plane, starting from the slice where it first shows up lateral to the internal capsule, surrounded by the other white matter tissues, and ending when it is no longer seen. Care was taken not to include blood vessels inferior to the putamen, the claustrum lateral to the putamen, or white matter tracts posterior to the putamen.

The nucleus accumbens was segmented out from the putamen when the internal capsule no longer separates the caudate nucleus and the putamen. Existing pipelines usually draw arbitrary lines to segment between the putamen and the accumbens, such as drawing a straight vertical line downwards from the lateral inferior tip of the internal capsule as suggested by the CMA guidelines. This is possibly due to the lower resolution of the structural image used in those segmentations. However, the anatomical boundaries between the putamen and the nucleus accumbens in the ICBM 2009b structural template are clearly more visible, and hence are directly used as references for segmentation.

## 3 Results

On the whole, the ALE meta-analyses revealed both cortical and subcortical clusters in the Go/No-Go, Stop-signal, and Think/No-Think tasks.

### 3.1 Cortical activations across the three tasks

Although the current effort emphasizes the role of the basal ganglia in stopping behaviour, we first briefly characterize cortical activations. Notably, although other GingerALE meta-analyses of motor response inhibition already have been published (e.g. Cai et al., 2014; Rae et al., 2014; Swick et al., 2011), the current analysis is the first to be published since the identification and correction of significant algorithmic bugs in the method used to correct for multiple comparisons that led to inadvertently liberal statistical thresholds being adopted (Eickhoff et al., 2017). The current activation maps therefore may be more circumscribed than those that have been previously reported owing to the newly corrected statistical corrections adopted.

**Fig. 3.1:**
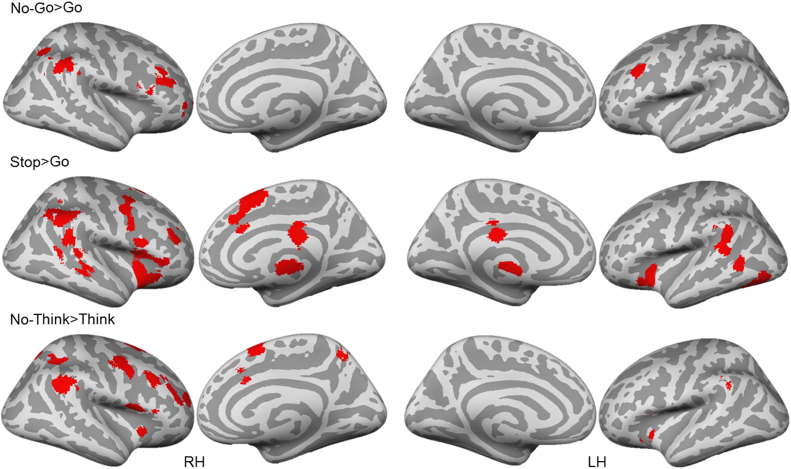
**Cortical activations from the task-specific meta-analyses.** All clusters are thresholded using cluster-level inference (*p*<.05, uncorrected *p*<.001, threshold permutations=1000).

As can be seen in Fig. 3.1, on the cortical level, preventing motor actions (Go/No-Go task) activated bilateral DLPFC and the right VLPFC, as well as regions in the right parietal lobes. Cancelling motor actions (Stop-signal task), on the other hand, activated the right DLPFC, VLPFC, and precentral gyrus. Action cancellation also activated bilateral insula, temporal and parietal regions, the cingulate gyrus and preSMA. These findings are generally consistent with those reported in previous meta-analyses (Cai et al., 2014, Rae et al., 2014, Swick et al., 2011), with several notable exceptions. First, Go-No-Go activations were less extensive than in prior reports (Swick et al., 2011) and did not include activation in either the left or right insula or left IFG. Second, although there was greater overall agreement between the current findings and those of Cai et al. (2014), our Stop-Signal analysis did not reveal activation in left DLPFC or frontal polar cortex. This difference may be attributable, however, to Cai et al.’s mixing of Stop-Signal and Go-No-Go studies into a common analysis, a possibility supported by presence of those missing activations in our Go-No-Go task analysis. These results were generated using cluster-level inference (p<.05, uncorrected p<.001, threshold permutations=1000). The cortical analysis also revealed that the Think/No-Think task activated the right DLPFC, VLPFC, cingulate gyrus, precentral gyrus, and the parietal lobe (including supramarginal/angular gyrus and intraparietal sulcus), as well as the left insula and supramarginal gyrus. The similarity of these activations to those observed in motor action stopping suggest the possible existence of domain-general cortical regions that contribute to memory and motor stopping, as suggested in prior work (Anderson et al., 2004, Depue et al., 2015). Indeed, all of the tasks activated the right DLPFC, VLPFC, and supramarginal/angular gyrus, in the right hemisphere.

**Fig. 3.2:**
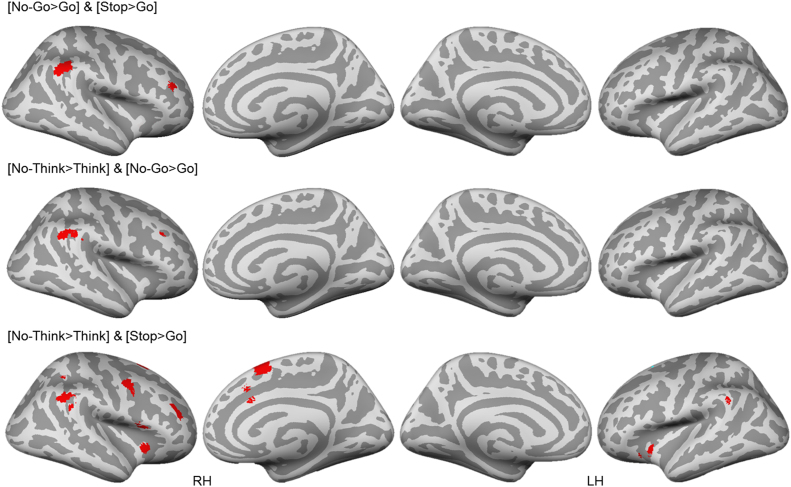
**Cross-task conjunction analysis.** All clusters are thresholded using cluster-level inference (*p*<.05, uncorrected *p*<.001, threshold permutations=1000).

To determine whether the foregoing characterizations of cross-task commonalities accurately represent inter-task relationships, we conducted a formal conjunction analysis. As can be seen in Fig. 3.2, action prevention (Go/No-Go) and Action Cancellation (Stop-Signal) tasks shared limited right lateralized activations in the right anterior DLPFC and the Supramarginal/Angular Gyrus. The existence of overlapping activations suggests that some elements of motor action cancellation and motor action prevention are shared, as one might expect, based on their classification as action stopping tasks. More interesting, however, was the differing relationship between memory inhibition (the Think/No-Think task) and the two motor response inhibition tasks. Whereas activations observed for memory inhibition overlapped extensively with action cancellation (Stop-signal task), overlap was more limited with action prevention (Go/No-Go). Specifically, action cancellation and memory inhibition shared right lateralized activations in the anterior DLPFC, posterior MFG, VLPFC, Insula, Angular/Supramarginal gyrus, Intraparietal Sulcus, Pre-SMA, and anterior cingulate. In contrast, action prevention and memory inhibition only shared activation in right angular/supramarginal gyrus and a very small region in right middle frontal gyrus that did not overlap with the region shared with action cancellation. These findings suggest that despite broad similarities of all three tasks in the involvement of right lateral prefrontal cortex, the spatial localisation of shared activations may provide additional information about the relative similarities between tasks. These data are consistent with the possibility that memory inhibition, at the cortical level, may have more in common with action cancellation than with action prevention.

### 3.2 Subcortical activations across the three tasks

On the subcortical level, all three tasks produced reliable clusters in the basal ganglia, suggesting that the basal ganglia are involved in both memory and motor inhibition and may be part of a supramodal network of inhibitory control. By qualitatively comparing the ALE results, we found a task-specific hemispheric asymmetry in the location of basal ganglia clusters. Specifically, significant activation clustering was localised to the left hemisphere for action prevention (Go/No-Go) task, whereas significant activation clustering was localised to the right hemisphere for action cancellation (Stop-signal) and memory inhibition (Think/No-Think) tasks. The following results sections will elaborate on findings in the basal ganglia. For a summary of all the basal ganglia results from the task-specific, conjunction, and contrast analyses, please see section 3.3.

#### 3.2.1 Comparing the cancellation and prevention of motor actions

On the whole, our analyses indicated that both action cancellation and prevention yielded clusters of activation in the basal ganglia. However, action cancellation yielded more spatially extensive clusters, which scarcely overlapped with the clusters from action prevention. The largely distinct localisation of basal ganglia clusters suggests that action cancellation and action prevention may engage distinct stopping processes that should not be assumed to be equivalent. This section illustrates these findings by detailing and comparing the clusters from the Stop-signal (cancellation) and Go/No-Go (prevention) tasks.

##### 3.2.1.1 Action cancellation engaged right basal ganglia structures

**Fig. 3.3:**
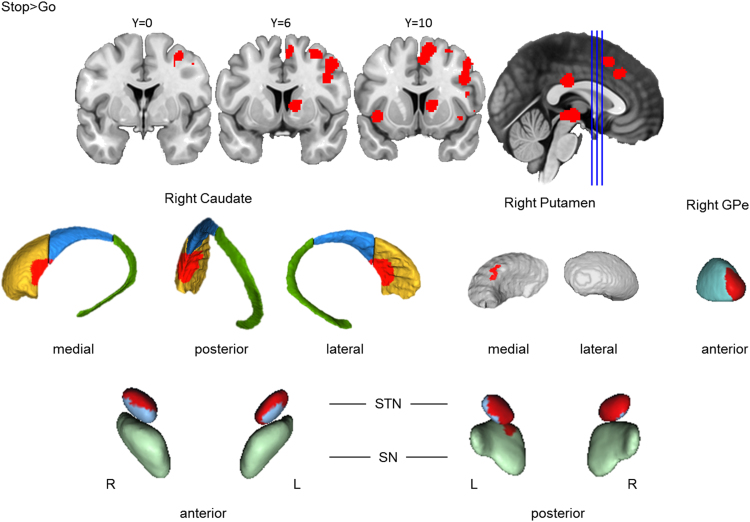
**Basal ganglia activation for action cancellation.** Top row: Clusters are presented on coronal slices of a high-resolution MNI atlas. Reference lines for the coronal slices are presented in the sagittal plane. Middle row: Clusters are displayed on high-resolution parcellations of the caudate, putamen, and external globus pallidus (GPe). Bottom row: Clusters are displayed on high-resolution parcellaions of the subthalamic nucleus (STN) and substantia nigra (SN). All clusters are thresholded using cluster-level inference (*p*<.05, uncorrected *p*<.001, threshold permutations=1000).

Across the 39 Stop-signal studies included in the analysis, cancelling a motor action yielded a consistent cluster in the right basal ganglia (Fig. 3.3). First, cancelling a motor action is associated with a cluster in the right centromedial striatum, primarily in the caudate head, spanning into the caudate body and the right anteromedial putamen. This cluster also extended to the right anterior GPe. Visual inspection suggests that the localisation of this cluster may coincide with the putative homologue of the region that receives DLPFC projections identified in the monkey literature (Haber and Knutson, 2010), a possibility consistent with the clear DLPFC activation observed during action cancellation in our cortical findings (Fig. 3.1). Second, significant clusters were also observed in the bilateral STN and left SN. The STN finding is compatible with the significant action cancellation role consistently attributed to this structure in previous literature (Aron and Poldrack, 2006). The SN activations are compatible with the dopaminergic modulation that is required by basal ganglia control mechanisms (Alexander and Crutcher, 1990). However, these activations from the STN and SN should be interpreted cautiously, as they are small in size and are neighboured by other subcortical nuclei, and our functional resolution is only as good as that of the ALE result maps. Thus, the reported activations may be driven by effects in neighbouring structures. Finally, cancelling a motor action also yielded a cluster in the ventral thalamus. The ventral thalamus is downstream to the basal ganglia and is specifically implicated in motor processes (Alexander et al., 1986).

##### 3.2.1.2 Action prevention reliably activated left putamen and GPe, but not caudate

**Fig. 3.4:**
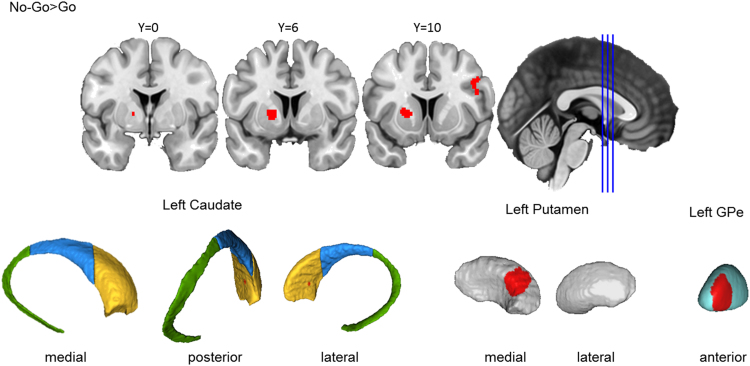
**Basal ganglia activation for action prevention.** Top row: Clusters are presented on coronal slices of a high-resolution MNI atlas. Reference lines for the coronal slices are presented in the sagittal plane. Bottom row: Clusters are displayed on high-resolution parcellations of the caudate, putamen, and external globus pallidus (GPe). All clusters are thresholded using cluster-level inference (*p*<.05, uncorrected *p*<.001, threshold permutations=1000).

Across the 30 Go/No-Go studies included in the analysis, preventing a motor action yielded a cluster in the left basal ganglia, including anterior putamen, spanning into anterior GPe, only touching on the medial wall of the caudate head (Fig. 3.4). The putamen involvement aligns with classic models of the cortico-basal ganglia circuit for motor control (Alexander et al., 1986). However, the absence of a caudate cluster during action prevention, as compared to action cancellation, suggests that these motor inhibition tasks may place different demands on neural mechanisms in the basal ganglia.

##### 3.2.1.3 Action cancellation and prevention showed no significant co-localisation in the basal ganglia

From the meta-analyses of individual task types, it is striking that action cancellation and prevention shared so few clusters in the basal ganglia, given that the Stop-signal and the Go/No-Go tasks are often used interchangeably to measure response inhibition. To formally test whether action cancellation and action prevention engaged similar basal ganglia structures, we computed a conjunction analysis between the Go/No-Go and Stop-signal tasks. No overlapping clusters were identified in the basal ganglia at the current threshold, although subthreshold clustering might exist in the Go/No-Go task (see contrast analysis in 3.2.1.4). It is unlikely that this lack of similarity between these tasks within the basal ganglia arises from insufficient statistical power, given the large number of studies included in the analysis. To the extent that interactions between cortical and basal ganglia structures are considered critical to the character of the stopping mechanism, these findings suggest that overlap at the cortical level between the Stop-signal and Go/No-Go tasks (Fig. 3.2) may not imply a common mechanism of action stopping.

Some have suggested that putative differences between the two tasks may be due to the variations in the administration of the Go/No-Go task (Levy and Wagner, 2011). Typically, the prepotency of the to-be-stopped motor response in the Go/No-Go and Stop-signal tasks is created by having frequent Go trials and infrequent No-Go or Stop trials (Wessel, 2017). However, some Go/No-Go studies have had equiprobable Go and No-Go trials, making the prepotency of the motor responses uncertain, and possibly undermining the necessity of inhibitory control. This is unlikely to be the case in our analysis, as only 9 out of 30 Go/No-Go studies used an equiprobable design, and another 2 with varying frequency of No-Go trials in different blocks of their task phase. The limited number of studies should not exert a strong influence on the results (Eickhoff et al., 2009, Eickhoff et al., 2011). To confirm this, we conducted a control meta-analysis including only Go/No-Go studies with infrequent No-Go trials (N=19), which revealed an identical cluster of activation in the left basal ganglia as the one reported in the original Go/No-Go meta-analysis (see Fig. 3.4). We then re-ran the conjunction between the Stop-signal and Go/No-Go tasks using the modified Go/No-Go sample (N=19). Again, we found no significant basal ganglia co-localisation of clusters between tasks. Hence, the null conjunction effect cannot be attributed to variation of prepotency in the Go/No-Go task.

##### 3.2.1.4 Action cancellation engaged the STN and SN significantly more than action prevention

**Fig. 3.5:**
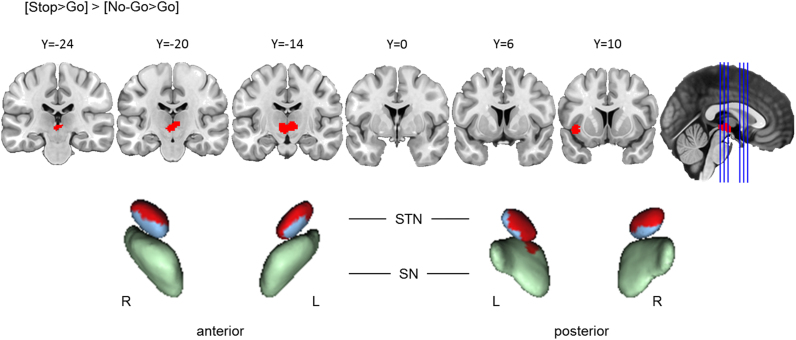
**Action cancellation reliably engaged STN and SN more than action prevention.** Top row: Clusters are presented on coronal slices of a high-resolution MNI atlas. Reference lines for the coronal slices are presented in the sagittal plane. Bottom row: Clusters are displayed on high-resolution parcellaions of the subthalamic nucleus (STN) and substantia nigra (SN). The contrast analysis was computed using the thresholded ALE images from the individual analyses. All clusters are thresholded at uncorrected *p*<.001, with the *p*-value permutations of 10,000 iterations, and the minimum cluster volume of 200 mm^3^.

Visual comparison of the clusters yielded by the Go/No-Go and Stop-signal tasks suggests that action cancellation engages both STN and SN, but that action prevention does not. To determine whether these differences are reliable, we computed a contrast analysis between the Stop-signal and Go/No-Go tasks. The results confirmed significantly greater clustered activation during action cancellation in bilateral STN and the left SN than during action prevention (Fig. 3.5), indicating a robust difference between the two stopping processes. However, as previously mentioned, these results should be interpreted cautiously given the small size of these nuclei and the possibility that the smoothed data might be influenced by activation in adjacent structures. Although in the task-specific meta-analyses action cancellation yielded clusters in the right caudate and action prevention did not, this observed difference was not statistically significant in the direct contrast analysis. This finding suggests that conclusions about the lack of right caudate involvement in action prevention should be tempered until firmer evidence of differential engagement across cancellation and prevention is clearly established (see Section 3.2.4 for data indicating that this observed difference in caudate involvement is better described as a difference in the spatial dispersion (clustering) of reported activation coordinates, as opposed to an absolute absence of reported coordinates per se).

##### 3.2.1.5 Action cancellation engaged the basal ganglia more extensively than action prevention

As mentioned previously, we observed significant clustering in the right striatum and GPe, bilateral STN, and left SN in the action cancellation task. By contrast, significant clustering was limited to the left striatum and GPe in the action prevention task. To quantify the extensiveness of basal ganglia clusters yielded by these tasks, we compared the total volumes of the clusters from the task-specific analyses. At our current threshold (cluster-level inference p<.05, uncorrected p<.001, threshold permutations=1000), cancelling a motor action yielded more extensive basal ganglia activation clusters overall (1120 mm^3^ in the right hemisphere and 216 mm^3^ in the left hemisphere) than preventing a motor action (864 mm^3^ in the left alone).

#### 3.2.2 Comparing memory and motor inhibition

Overall, our analysis revealed that memory inhibition yielded consistent activation clusters in the right basal ganglia, but not in the left. Importantly, when we compared the basal ganglia activation clusters observed for memory and motor inhibition, we found that memory inhibition yielded clusters that were highly similar to those involved in action cancellation, but not to those involved in action prevention. This section delineates the basal ganglia clusters observed for memory inhibition, and compares them with those yielded by action cancellation and action prevention.

##### 3.2.2.1 Memory inhibition engaged right caudate, putamen, and GPe

**Fig. 3.6:**
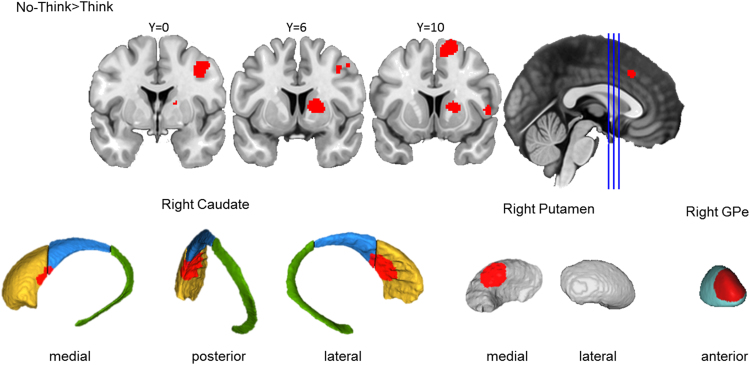
**Memory inhibition engaged the right basal ganglia.** Top row: Clusters are presented on coronal slices of a high-resolution MNI atlas. Reference lines for the coronal slices are presented in the sagittal plane. Bottom row: Clusters are displayed on high-resolution parcellations of the caudate, putamen, and external globus pallidus (GPe). All clusters are thresholded using cluster-level inference (*p*<.05, uncorrected *p*<.001, threshold permutations=1000).

Across the 16 Think/No-Think studies included in the analysis, memory inhibition yielded a significant activation cluster in the right basal ganglia. This cluster was primarily located in the caudate head, spanning into caudate body, anterior putamen, and anterior GPe (Fig. 3.6). This cluster is highly similar to the one yielded by action cancellation in the centromedial striatum. Together with the consistent activation in the DLPFC reported in Fig. 3.1, these results suggest that a similar DLPFC-basal ganglia control mechanism may be engaged by both memory inhibition and action cancellation. Memory inhibition yielded a more extensive basal ganglia activation cluster in the right hemisphere (1648 mm^3^) than did action cancellation (1120 mm^3^).

##### 3.2.2.2 Memory inhibition and action cancellation engaged right caudate, putamen, and GPe

**Fig. 3.7:**
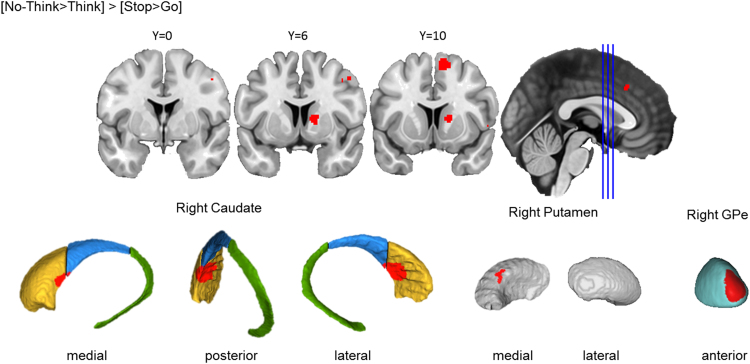
**Spatial Co-localisation of memory inhibition and action cancellation in basal ganglia subregions.** Top row: Clusters are presented on coronal slices of a high-resolution MNI atlas. Reference lines for the coronal slices are presented in the sagittal plane. Bottom row: Clusters are displayed on high-resolution parcellations of the caudate, putamen, and external globus pallidus (GPe). All clusters are thresholded using cluster-level inference (*p*<.05, uncorrected *p*<.001, threshold permutations=1000).

To formally test whether the basal ganglia activation clusters generated by memory inhibition and action cancellation overlapped, we examined the conjunction analysis between the ALE maps for the Think/No-Think and Stop-signal meta-analyses. Critically, within the basal ganglia, the results demonstrated that both tasks activated the right caudate head/body, anterormedial putamen, and anterior GPe (Fig. 3.7). Specifically, at the cluster-corrected threshold, the conjunction cluster resulted in an extensive overlap (552 mm^3^) with the task-specific Think/No-Think and Stop-signal basal ganglia clusters, constituting 33% of the basal ganglia cluster volumes activated by memory inhibition, and 49% of those activated by action cancellation in the right hemisphere, or 41% overall. When considered together with the shared cortical activations in the right anterior and posterior DLPFC (Fig. 3.2), these findings suggest that the putative DLPFC-basal ganglia pathway may serve a supramodal inhibitory control function across memory and motor domains.

##### 3.2.2.3 Memory inhibition and action prevention did not reliably co-localise in the basal ganglia

Intriguingly, memory inhibition and action prevention did not seem to share basal ganglia activation clusters from the individual maps, as the first yielded a cluster exclusively located in right basal ganglia, and the latter, a cluster exclusively in left basal ganglia. To quantitatively verify this observation, we examined the conjunction analysis between the Think/No-Think and Go/No-Go tasks. As suggested by the individual meta-analyses, these tasks did not share common activations in the basal ganglia at our current threshold. As with the stop-signal task, we also examined whether the failure to detect conjunction effects may be due to variation of prepotency in the Go/No-Go task. This was not the case: when we re-analysed the conjunction between the Think/No-Think and Go/No-Go tasks using the modified Go/No-Go sample (studies with offset ratios of Stop and Go trials; N=19), we were unable to detect significant basal ganglia co-localised clusters between the Think/No-Think and Go/No-Go tasks. The lack of shared activations in the basal ganglia accords well with the minimal overlap with action prevention and memory inhibition at the cortical level (Fig. 3.2). These findings suggest that although memory inhibition and action prevention engage moderately overlapping common cortical regions (e.g. right angular gyrus), they seem to recruit distinct processes in the basal ganglia. These findings are consistent with the possibility that memory inhibition in the Think/No-Think task primarily requires cancellation of memory retrieval.

##### 3.2.2.4 Memory inhibition engaged basal ganglia subregions more reliably than motor inhibition

**Fig. 3.8:**
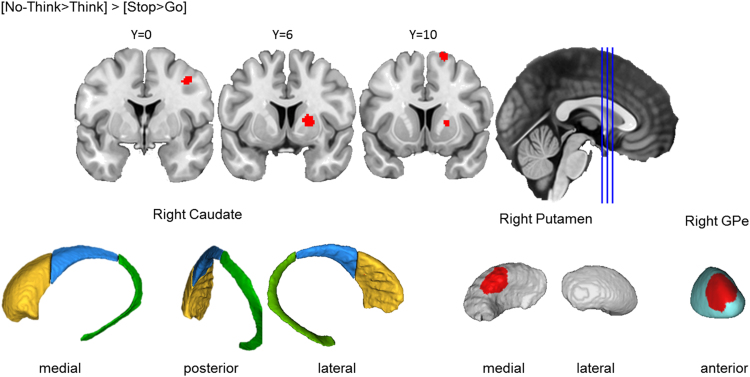
**Memory inhibition engaged putamen and GPe more reliably than action cancellation.** Top row: Clusters are presented on coronal slices of a high-resolution MNI atlas. Reference lines for the coronal slices are presented in the sagittal plane. Bottom row: Clusters are displayed on high-resolution parcellations of the caudate, putamen, and external globus pallidus (GPe). All clusters are thresholded using cluster-level inference (*p*<.05, uncorrected *p*<.001, threshold permutations=1000).

To quantify the differences between memory inhibition, action cancellation, and action prevention, we computed contrast analyses between the Think/No-Think and Stop-signal tasks, and between the Think/No-Think and Go/No-Go tasks. Comparing the Think/No-Think and Stop-signal tasks, although both tasks yielded activation clusters in similar regions in the right basal ganglia, memory inhibition engaged the right anteromedial putamen and anterior GPe more than did action cancellation (Fig. 3.8). This finding is intriguing as the putamen is usually construed as part of the motor circuit (Alexander et al., 1986). However, recent studies have shown putamen activations thought to reflect the interaction between memory, action and reward (Koster et al., 2015), indicating that the putamen is not functionally limited to involvement in motor control tasks. Indeed, Seger et al. (2011) reported evidence for effective connectivity between the putamen and the posterior hippocampus, providing at least one precedent for a potentially important role of the putamen in hippocampal interactions.

**Fig. 3.9:**
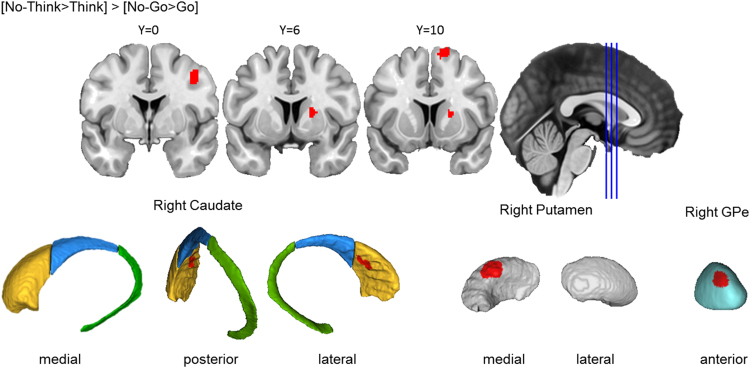
**Memory inhibition engaged caudate, putamen, and GPe more reliably than action prevention.** Top row: Clusters are presented on coronal slices of a high-resolution MNI atlas. Reference lines for the coronal slices are presented in the sagittal plane. Bottom row: Clusters are displayed on high-resolution parcellations of the caudate, putamen, and external globus pallidus (GPe). All clusters are thresholded using cluster-level inference (*p*<.05, uncorrected *p*<.001, threshold permutations=1000).

When we compared the Think/No-Think and Go/No-Go tasks (Fig. 3.9), memory inhibition engaged more clustered activity in the right anteromedial putamen and anterior GPe than did action prevention. This echoes the contrast between memory inhibition and action cancellation. In addition, memory inhibition yielded stronger evidence of clustered activations in the right caudate head. The caudate is usually construed as part of the executive function circuit (Alexander, 1986; Seger, 2013). It is possible that inhibiting memory retrieval requires more active control processes especially when intrusions take place, whereas action prevention can be achieved by low-level associative learning.

##### 3.2.2.5 Action cancellation engaged STN more reliably than memory inhibition

**Fig. 3.10:**
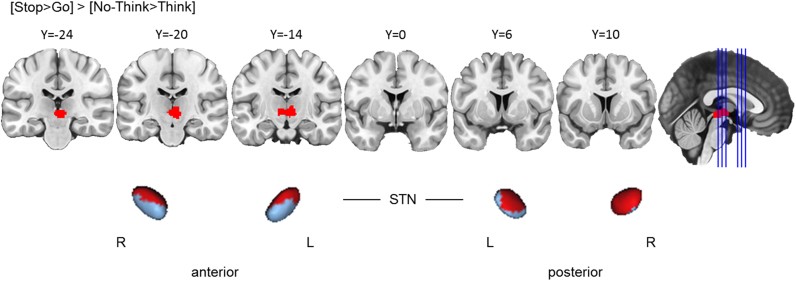
**Action cancellation engaged STN more reliably than memory inhibition.** Top row: Clusters are presented on coronal slices of a high-resolution MNI atlas. Reference lines for the coronal slices are presented in the sagittal plane. Bottom row: Clusters are displayed on high-resolution parcellaions of the subthalamic nucleus (STN). The contrast analysis was computed using the thresholded ALE images from the individual analyses. All clusters are thresholded at uncorrected *p*<.001, with the *p*-value permutations of 10,000 iterations, and the minimum cluster volume of 200 mm^3^.

We also examined which regions yielded greater activation clustering during action cancellation than by memory inhibition. Our individual analyses had revealed bilateral STN and left SN activation clusters in action cancellation but not in memory inhibition. To formally test these differences, we computed a contrast analysis between the Stop-signal and Think/No-Think tasks. Our results revealed that action cancellation yielded reliably greater activation clustering in bilateral STN and ventral thalamus than did memory inhibition (Fig. 3.10). Again, care should be taken in interpreting meta-analytic activations for these fine subcortical structures, as they may be influenced by effects in neighbouring structures.

#### 3.2.3 Summary of ALE results in the basal ganglia

**Fig. 3.11:**
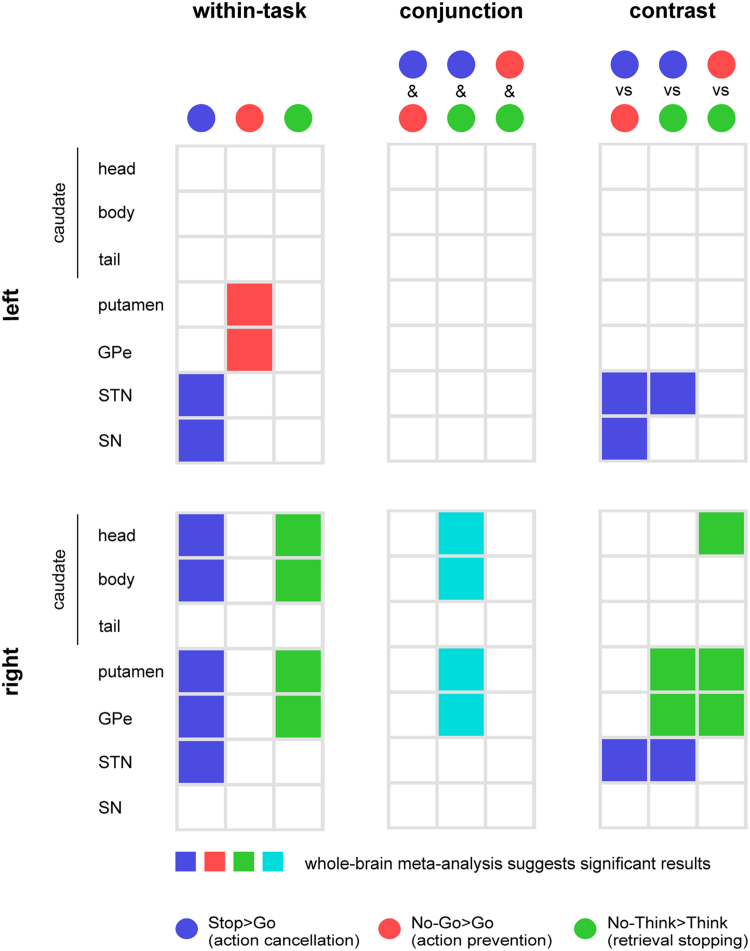
**Basal ganglia activations in the task-specific, conjunction, and contrast analyses.** The left column shows basal ganglia activations from the task-specific meta-analyses, colour-coded by task contrasts (Blue=Stop>Go, Red=No-Go>Go, and Green=No-Think>Think). The middle column shows the conjunction analyses. Activations shared by two tasks are presented in the mixed colour based on the colours that we used to represent the individual tasks. The right column shows basal ganglia activations from the contrast analyses, with the colours denoting task-specific activity. For example, bilateral STN was activated more strongly in the Stop>Go contrast (blue) than the No-Go>Go and No-Think>Think contrasts. The top panel summarises activations in the left basal ganglia structures, while the bottom panel summaries those in the right.

The basal ganglia ALE results are summarised in Fig. 3.11**,** including activations from the task-specific, conjunction, and contrast analyses. This summary shows that whereas the Go/No-Go task primarily engages the left putamen and GPe, the Stop-signal and Think/No-Think tasks primarily engage the right caudate, putamen, and GPe. In other words, our findings suggest that the cancellation of actions and thoughts engages similar basal ganglia structures, and that these are distinct from the basal ganglia structures engaged in the prevention of actions. This does not necessarily mean that none of the action prevention studies activated the same basal ganglia structures as action cancellation and memory inhibition. However, this does suggest that, if action prevention studies did activate the same structures (in particular, the right basal ganglia), the activation peaks were not sufficiently spatially clustered to be detected with the ALE algorithm. We explore these considerations further in Section 3.2.4.

#### 3.2.4 Visualisation of basal ganglia activation peaks from the included studies

**Fig. 3.12:**
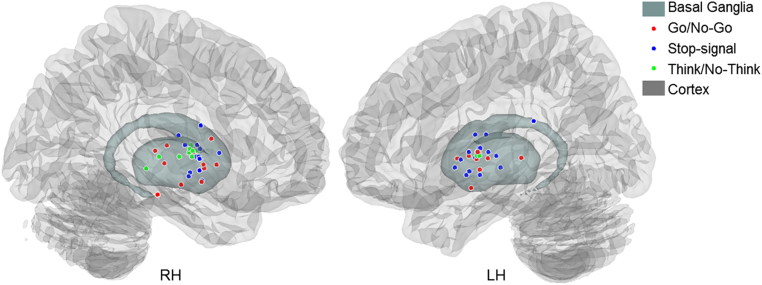
Peak coordinates from the basal ganglia activations in the Go/No-Go, stop-signal, and Think/No-Think tasks.

By definition, the ALE analyses are sensitive to common clusters of activation across studies. Activation peaks that are more spatially dispersed, either because a particular cognitive function is not associated with localised activation, or because the included studies are few in number, therefore might not be detected as common across studies. To explore this possibility further, we visualised the spatial distribution of peak activations across studies on 3D renders of the pre-defined basal ganglia ROIs (Fig. 3.12), and counted the number of coordinates located in the left and right basal ganglia (Table 3.1). Of particular interest is whether any Go/No-Go studies activated the right basal ganglia, because we did not find any common clusters in the right basal ganglia across the Go/No-Go task (action prevention) and either of the other tasks (Stop-signal and Think/No-Think). Examining the peak coordinates from the basal ganglia activations in the original studies showed that the basal ganglia are indeed activated in all three tasks, and that there are overlaps between the basal ganglia activations from the memory and motor inhibition tasks. However, several novel observations emerge from these summaries. First, although our ALE analysis only revealed significant clusters of activation in the left basal ganglia for the Go/No-Go task, there are, interestingly, equally many peak coordinates that appear in both the left and right hemispheres. The key difference between the left and right basal ganglia activations seems to be the somewhat greater dispersion of coordinates in the right hemisphere, reducing the apparent clustering. Second, although the ALE analysis only demonstrated significant clusters of activation in the right basal ganglia for the Stop-signal task, there are equally many coordinates in the left and right hemispheres. The coordinates seemed more dispersed in the left hemisphere, again reducing the apparent clustering. Finally, memory inhibition qualitatively appears to be more right lateralised than the other tasks, consistent with the impression offered by ALE. A more precise characterisation of task differences in the spatial distribution of activations across sub-regions is limited by the moderate number of coordinates available in this dataset.

**Table 3.1:** Number of studies reporting basal ganglia coordinates in the left and right hemispheres from the Go/No-Go, stop-signal, and Think/No-Think tasks.

	Left Hemisphere			Right Hemisphere		
	Studies	Coordinates	% of total studies	Studies	Coordinates	% of total studies
Go/No-Go	7	9	23%	7	10	23%
Stop-signal	10	15	26%	13	15	33%
Think/No-Think	3	3	19%	8	10	50%

## 4 Discussion

The current investigation examined the potential existence of common mechanisms in the basal ganglia that underlie the inhibition of actions and thoughts. Although the basal ganglia have an established role in motor inhibition, whether and how this structure is involved in memory inhibition remains unexplored. To address these issues, we conducted a set of meta-analyses using fMRI data from the Go/No-Go, Stop-signal, and Think/No-Think tasks. Whereas the first two tasks require inhibiting motor actions, the last task requires inhibition of episodic memory retrieval. After examining the ALE maps for each task, we computed conjunction and contrast analyses to formally examine the similarities and differences between the locations of significant basal ganglia clusters recovered in each task. Moreover, because the ALE analysis may be more sensitive to spatially clustered than spatially distributed activations, we also examined basal ganglia peak coordinates from the studies included to illustrate the prevalence and dispersion of basal ganglia activations yielded by each task. We localised the observed basal ganglia clusters as precisely as possible by manually segmenting the striatal sub-regions from a high-resolution template brain, including the caudate head, body, and tail, and the putamen. This is the first segmentation to our knowledge that has individual compartments of the striatum at this level of spatial resolution. Our key observations and their implications are discussed below.

On the cortical level, our motor inhibition meta-analyses resembled prior meta-analytic findings (Cai et al., 2014, Rae et al., 2014, Swick et al., 2011), including activations in the right DLPFC, VLPFC, and angular/supramarginal gyrus for both Stop-signal and Go/No-Go tasks. Notably, however, the spatial extent of meta-analytic activations was reduced overall, owing to our use of a corrected version of GingerALE which corrects for multiple comparisons accurately (Eickhoff et al., 2017). Moreover, in contrast to prior meta-analyses (e.g. Swick et al., 2011) which found evidence for bilateral insula activation in the Go/No-Go task, we did not. Our memory inhibition meta-analysis also revealed activations in the right DLPFC and VLPFC, in addition to the anterior cingulate cortex, pre-SMA, and right parietal regions. These findings are broadly consistent with the possibility that stopping engages a supramodal cortical network, irrespective of whether one is stopping actions or thoughts. Nevertheless, despite these broad similarities, individual conjunctions between tasks revealed differences in the extent to which memory inhibition shared activations with action prevention versus action cancellation. Whereas memory inhibition shared activations with action cancellation in right anterior and posterior DLPFC, VLPFC, Insula, ACC, Pre-SMA, angular/supramarginal Gyrus, and intraparietal sulcus, it's overlap with motor action prevention was considerably more limited, especially in the right prefrontal cortex. This difference suggests that memory inhibition may engage stopping processes that require cancellation to a greater extent than it requires retrieval prevention. Alternatively, the Go/No-Go task may simply be less demanding than the other two tasks, yielding less prefrontal activation overall, and correspondingly less possible overlap with action cancellation and memory inhibition.

On the subcortical level, although we observed basal ganglia clusters in all three tasks, the specific localisation of these clusters differed. Strikingly, the Go/No-Go and Stop-Signal tasks – two of the most widely studied forms of motor stopping that are often assumed to engage similar functions – showed clusters in different basal ganglia regions. Whereas the Go/No-Go task consistently activated the left anterior putamen (spanning into anterior GPe), the Stop-signal task yielded more extensive right-lateralised spatial clusters of activation mainly in the caudate head/body, anterodorsal putamen, anterior GPe. A formal conjunction analysis revealed that overlap between the activation clusters observed in these tasks was not statistically significant. These findings hold both when we included all 30 Go/No-Go studies, and when we excluded those with equiprobable Go and No-Go trials. To ensure that inhibitory processes are taking place, future Go/No-Go studies should always have infrequent No-Go trials in the design (Levy and Wagner, 2011, Wessel, 2017). The differing localisations of these clusters may be very important for two reasons. First, distinct basal ganglia structures constitute different coordinating pathways supporting the prevention, initiation, and termination of motor or cognitive processes (Alexander and Crutcher, 1990, Graybiel, 2005, Scimeca and Badre, 2012). Second, cortical and subcortical structures project topographically to the basal ganglia (Haber, 2003, Winn et al., 2009). Therefore, differently localised activation clusters, such as those observed here, could indicate different computational functions (Alexander and Crutcher, 1990, Haber et al., 2006, Lanciego et al., 2012, Seger, 2013). These observations converge with recent findings suggesting that the Go/No-Go and Stop-signal tasks may differ in important respects, including the underlying cognitive processes engaged (Schachar et al., 2007, Verbruggen and Logan, 2008), cortical regions recruited (Dalley et al., 2011), their electrophysiological markers (Johnstone et al., 2007) and neuropharmacological underpinnings (Eagle et al., 2008). These differences may arise because the Go/No-Go task primarily requires the prevention of a motor action from taking place, whereas the Stop-signal task requires cancelling an emerging or ongoing motor process. Thus, the current analysis of activation clusters support the view that despite their similarity as motor stopping procedures, these tasks may tap different control processes and should not be treated equivalently.

After comparing the Go/No-Go and Stop-signal tasks, we examined whether the basal ganglia were involved in stopping memory retrieval. Interestingly, we found that, like stopping actions, stopping thoughts also engages the basal ganglia. Memory inhibition in the Think/No-Think showed a consistent cluster of activation in the right caudate head/body, anterodorsal putamen, and anterior GPe. This cluster of activations was exclusively right lateralised, and was more spatially extensive than the analogous clusters from the motor stopping tasks. This clearly indicates that basal ganglia structures play an important role in stopping retrieval, perhaps akin to its role in stopping actions. This commonality raises the possibility that basal ganglia structures are involved in stopping in a more general way than is usually assumed in research on motor inhibition. A similar supramodal hypothesis was discussed by Aron (2007), though with a more specific focus on interactions between VLPFC and the subthalamic nucleus, rather than the basal ganglia more broadly.

Although both memory and motor inhibition activated the basal ganglia, the pattern of activations in that structure provides converging evidence that memory inhibition in the Think/No-Think task may be more similar to action cancellation in the Stop-signal task than it is to action prevention in the Go/No-Go task. Consistent with their strong overlap at the cortical level (Fig. 3.2), our conjunction analysis revealed strong overlap between the activation clusters observed for memory inhibition and action cancellation, including the right caudate head/body, anterior putamen, and the anterior GPe. Critically, the conjunction cluster between memory inhibition and action cancellation in the right hemisphere constituted 33% of the voxels activated by memory inhibition, and 49% of those activated by action cancellation in the right hemisphere (or 41% when considering both hemispheres). These findings suggest that the particular basal ganglia regions observed here might play a computational role in cancelling a process, irrespective of whether that process involved motor action. Action cancellation, however, did engage bilateral STN and ventral thalamus more reliably than did memory inhibition. It is possible that these regions are uniquely required for cancelling a motor response, as the ventral thalamus is typically construed as the downstream target of the basal ganglia during motor control (Alexander et al., 1986). The STN is also shown to be integral for cancelling a motor action (Aron and Poldrack, 2006), although which specific pathway the STN engages (either the hyperdirect or the indirect pathway) remains unresolved. However, given their small size and the lack of attention to these structures in the literature on memory inhibition, their activity during memory inhibition tasks might not have been consistently reported, even if it occurred. Future studies of memory inhibition should specifically examine the role of the STN in this process. More generally, connectivity analyses could be conducted to investigate the network dynamics between the basal ganglia structures to isolate the particular basal ganglia mechanisms underlying the inhibition of memory retrieval.

Despite the foregoing between-task differences in the STN activation clustering, the overall similarity between the clusters observed for memory inhibition and action cancellation in the striatum and GPe suggests that inhibiting thoughts may require active cancellation. This observation argues against the possibility that people prevent retrieval of an unwanted item by simply directing the retrieval process to distracting thoughts, or, instead, by passively failing to engage retrieval. Rather, the recruitment of cancellation-related striatal processes suggests that retrieval is being actively stopped. This interpretation converges with findings indicating that the engagement of inhibitory mechanisms during retrieval stopping is particularly robust when memories intrude into awareness and need to be purged (Levy and Anderson, 2012, Benoit et al., 2014). Using trial-by-trial intrusion reports, it has been found that intrusions elicit greater recruitment of right prefrontal cortex (Benoit et al., 2014) and greater down-regulation of hippocampal activity (Levy and Anderson, 2012), compared to trials without intrusions. The current findings suggest that retrieval cancellation may be key to overcoming intrusions. In contrast, we observed no overlap in activation clusters between memory inhibition and action prevention from the ALE analyses. These findings are consistent with the possibility that different basal ganglia regions contribute to distinct cancellation and prevention-related sub-processes, and that cancellation is not tied uniquely to motor action, but rather may be supramodal. To establish these conclusions more firmly, however, requires that we move beyond mere co-localisation of activations to study dynamic interactions of these basal ganglia structures with other elements of the putative control network, under conditions of cancellation and prevention.

Our findings raise questions about the connectivity underlying these dynamic interactions. Of particular interest is the connectivity of these basal ganglia regions with other putative supramodal areas associated with inhibitory control (e.g., DLPFC, VLPFC), and also with domain-specific regions involved in memory and action, such as the hippocampus and the primary motor cortex (M1) respectively. For example, in our meta-analyses, by localising clusters within the basal ganglia, we observed that all of the Go/No-Go, Stop-signal, and Think/No-Think tasks recovered clusters in the centromedial striatum, including the caudate head/body, spanning across the internal capsule into medial putamen. This cluster roughly coincides with the region identified by Haber et al. (2006) that receives projections from the DLPFC (areas 9/46). Although much care is needed when comparing anatomical landmarks across species, Neggers et al. (2015) presented evidence based on diffusion imaging that the frontostriatal projections from anterior prefrontal cortex are more similar between humans and macaque monkeys than those from posterior frontal regions such as the frontal eye field (FEF) and M1. Since the DLPFC is thought to play important roles in stopping actions and thoughts (Anderson et al., 2004; Anderson and Hanslmayr, 2015; Depue et al., 2010, Depue et al., 2015), and since this general belief was strongly confirmed in our meta-analytic conjunction analysis for action cancellation and memory inhibition (Fig. 3.2), this putative DLPFC-striatal pathway could be a candidate through which memory and motor inhibition are achieved. This possibility must await further confirmation.

Despite its similarity to action cancellation, the memory inhibition cluster extended to parts of the right putamen and GPe more than did motor stopping in general. It is unclear what functions these potentially memory-inhibition-specific activations of putamen and GPe may be performing, or whether these functions are unique to this process or simply a more robust and spatially extensive engagement of putamen processes observed during action cancellation. The possibility that parts of the putamen may serve functions specific to memory control should be considered. It is worth noting, for example, that although the putamen is often seen as a motor structure (Alexander et al., 1986), recent evidence suggests that it is involved in cognitive processes such as working memory (Voytek and Knight, 2010), episodic memory encoding (Sadeh et al., 2011), and cognitive control (Badre and Wagner, 2007), and both neuroimaging and computational modelling suggest that the basal ganglia play critical roles in memory processes (Gruber et al., 2006, O'Reilly and Frank, 2006, Scimeca and Badre, 2012). Indeed, Koster et al. (2015) also found that the putamen is significantly activated in the interaction between memory, action, and reward. Specifically, participants learned four different categories of objects, each indicating whether the participants should respond to a following visual stimulus, and whether the correct action/inaction would lead to a reward or avoid a loss. They found that activity in the right dorsal putamen significantly predicted memory retrieval when the associated action/inaction led to the expected, but not to the unexpected level of reward. Although these related findings do not speak to a role of the putamen in memory inhibition, they do indicate that this structure interacts with the medial temporal lobes during memory tasks, providing precedent for such a role. The circuitry underlying this potential contribution to memory inhibition remains to be identified.

On top of the established network of motor control involving the basal ganglia, several authors have discussed potential interactions between the basal ganglia and the hippocampus. While some found that the basal ganglia and the hippocampus may be largely independent from each other (Döller et al., 2008), others have suggested more complex relationships between the two systems during memory functions. On the one hand, basal ganglia and hippocampal processes may be competitive in nature, such that increased activation in one structure is associated with decreased activation in the other (Dagher et al., 2001, Poldrack and Packard, 2003). Poldrack and Rodriguez (2004) reviewed evidence for the competition between the hippocampal and basal ganglia systems in classification learning, and proposed that the competition may be modulated by task demands and behavioural success. Rodriguez and Poldrack (2003) re-analysed a classification learning dataset wherein participants performed a weather prediction task. In this task, participants performed on-line learning where they associated visual stimuli with weather categories. Using structural equation modelling, they identified that the competitive interaction between the basal ganglia and the medial temporal lobe is mediated by the prefrontal cortex. This work provides evidence against direct interactions between the basal ganglia and the hippocampus, at least in the context of Rodriguez and Poldrack's classification task.

Despite this evidence that the basal ganglia and the hippocampal systems are independent or interact through the prefrontal cortex, other evidence has suggested that the basal ganglia and hippocampus may interact in other ways. For example, Sabatino and colleagues found evidence that basal ganglia activity influences hippocampal oscillations. Specifically, whereas caudate stimulation appeared to influence the hippocampal theta rhythm by inhibiting the hippocampal spikes (La Grutta et al., 1985, Sabatino et al., 1985), pallidal stimulation triggered enhanced epileptiform activity, inducing generalised seizure activity (Sabatino et al., 1986). Berke et al. (2004) also found entrainment of ventral/medial striatal neurons to the hippocampal theta in rats. Moreover, using Granger Causal Modelling on fMRI data, Seger et al. (2011) found evidence for effective connectivity from the putamen to both the caudate and posterior hippocampus, as well as from posterior hippocampus to the caudate. These interactions were observed in two tasks. One was a weather prediction task, where participants learned on-line whether a visual stimulus was meant to predict rain or sunshine. The other was a subjective judgement task, wherein the participants rated whether their weather categorisation was based on memories or guesses. The foregoing findings raise the possibility that the basal ganglia may exert a controlling influence on target structures in both memory and motor inhibition. In the case of memory inhibition, this controlling influence may arise through complex polysynaptic interactions with the hippocampus. Further research is needed to elucidate how these interactions might be achieved.

Ultimately, determining the extent to which a supramodal cancellation process truly exists will depend on whether intrinsic basal ganglia pathways are engaged in similar ways for memory and motor inhibition tasks, including the direct, indirect, and hyperdirect pathways. Unfortunately, meta-analytic activations do allow us to determine which specific pathways are required by these tasks. For example, increased striatal activity may imply engagement of either the direct or indirect pathway, or an interaction between the two. Similarly, increased STN activity may indicate engagement of either the hyperdirect or the indirect pathway. Despite these limits on our data, it is worth considering how a supramodal cancellation process might emerge from these pathways. In one recent effort (Schroll and Hamker, 2013) analysed a range of computational models characterising the cognitive and motor functions of the basal ganglia with possible contributions from these interacting pathways. Specifically, global blocking of activations, such as premature-response prevention and working memory updating, may be modulated by the hyperdirect and the indirect pathways; response inhibition/deferral and working memory gate closing may be modulated by the interaction between the direct and the short indirect pathways. Some of the proposals developed in this analysis might be extended to characterize how the basal ganglia are engaged to suppress retrieval from episodic memory, and the precise manner in which this process resembles action cancellation.

Although we sought to localise basal ganglia clusters in memory and motor inhibition tasks, our approach is not without caveats. For example, Wager et al. (2007) discussed a few limitations in Activation Likelihood Estimation (ALE). Due to the coordinate-based nature of the ALE algorithm, the analysis only considers the peak coordinates reported in each study, but not the extent of each cluster of activation where the peaks lie. In addition, the peak coordinates may be influenced by the specific methods used in each study (e.g., thresholding, smoothing, registration and normalisation). Most of the studies included in the current study adopted standard preprocessing methods from widely used neuroimaging software (e.g., SPM, FSL, AFNI), including slice-time correction, motion correction, normalisation, and smoothing. There were, however, variations in approach. For example, the smoothing kernel ranged from 3 mm to 12 mm and a few studies also used customised methods (Supplement). Moreover, a number of studies conducted first-level analyses in native space (Supplement), and later normalised the contrast images to standard templates. These variations necessarily limit the spatial precision we can attribute to the current findings and should be considered in interpreting the data.

Furthermore, the ALE activation maps, rightfully, model the spatial uncertainty of the reported peak coordinates from each study, which introduces a certain level of spatial smoothness. These factors also recommend caution when drawing conclusions about the precise localisation of our observed activations, given limitations on spatial resolution inherent to the meta-analytic method. Reporting bias is also a consideration, because some researchers may choose to omit activation peaks that do not fit prior expectations for a task, especially if the spatial extent of the activation is small, as would be true for some of the structures of key interest within the basal ganglia. These caveats have led some to argue that results from coordinate-based meta-analysis should be treated as an integration of existing knowledge instead of the absolute truth (Rottschy et al., 2012), as more accurate and complete information would require an image-based meta-analysis or ‘mega-analysis’ (Salimi-Khorshidi et al., 2009).

One final caveat, applicable to this and all other ALE meta-analyses, concerns how to interpret lack of significant clusters in a structure of interest. One hand, failing to find a significant cluster for a particular task may indicate that the structure is genuinely not engaged in the task. On the other hand, because the ALE algorithm seeks to identify clusters of activation, lack of a significant cluster may also be consistent with the presence of more dispersed activation peaks that fail to constitute a significant cluster. Indeed, the visualisation and counts of activation peaks in the left and right basal ganglia show clearly that there exist activations in basal ganglia structures in both hemispheres, especially for our two motor stopping tasks (see Fig. 3.12 and Table 3.1). Thus, whether one should interpret the differently lateralized clusters for action prevention and cancellation derived from ALE as indicating a meaningful task dissociation depends on the assumption that spatially clustered activations are more meaningful than those that are more dispersed. Regardless of the method of analysis, however, memory inhibition in the Think/No-Think task appeared to yield more spatially concentrated activations predominantly lateralised to the right basal ganglia. Due to the moderate number of coordinates available in current studies, however, quantitative examination of task-related differences in the spatial distribution of coordinates across sub-regions of the basal ganglia must await future studies.

Despite these limitations, our meta-analyses have provided the first meta-analytic evidence that memory and motor inhibition (action cancellation in particular) engage overlapping regions within the basal ganglia. These patterns suggest that similar frontostriatal pathways may be involved when people stop thoughts or actions. Moreover, by localising the observed clusters within our high-resolution manual segmentation of striatal subregions, we hope that our results can serve as a useful reference against which the results of future studies may be compared.

## 5 Conclusions

The current meta-analyses demonstrate that the basal ganglia are consistently activated in the inhibition of both actions and thoughts. This basic finding is broadly congruent with recent literature indicating that the basal ganglia are not merely involved in motor control, but also in higher-level cognitive processes, such as memory. Importantly, however, the surprising similarity of memory inhibition to action cancellation more than action prevention suggests that the nature of the stopping processes that are recruited may dictate the localisation of basal ganglia activity more so than does task domain, at least for the tasks we studied. Our data indicate that, during cancellation, similar cortical and basal ganglia regions are engaged, irrespective of the domain of the process that is controlled, consistent with the possibility of a supramodal cancellation process. Meanwhile, the differences in activation clusters between the Go/No-Go and Stop-signal tasks suggest that they may engage different stopping processes and that it may imprudent to treat these tasks equivalently. However, it bears emphasis that the current ALE meta-analysis is more sensitive to clustered activations than to dispersed ones. The inference that motor cancellation and motor prevention are distinctly localised in these data depends on the assumption that highly clustered activations (as detected by ALE) provide a more informative signature of functional specialization in the basal ganglia than more dispersed activations would, an assumption that deserves to be critically examined when more data is available. Importantly, future studies should characterise the specific basal ganglia engagement in memory and motor inhibition and investigate how the frontal, basal ganglia, and domain specific target regions (e.g., motor cortex and hippocampus) interact to perform specific stopping processes in different task domains. Extending the study of the role of the basal ganglia in inhibitory control to measure the stopping of both actions and thoughts will provide a valuable source of constraint on hypotheses about the computational functions that the basal ganglia perform.

## Footnotes

1 It might be useful to note that the observed spatial dispersion of activation foci reflects true dispersion as well as measurement noise, i.e. studies with smaller sample sizes are expected to yield more spatially distributed foci than studies with larger sample sizes. However, as long as study sample sizes do not differ substantially across the different tasks, we assume that observed differences in spatial dispersion between tasks can largely be attributed to true differences in dispersion between tasks.
