# Supplementary material for: A supramodal role of the basal ganglia in memory and motor inhibition: Meta-analytic evidence
Source: Neuropsychologia. 2018 Jan 8;108:117–34. doi: 10.1016/j.neuropsychologia.2017.11.033 (PMC5759998; doi:10.1016/j.neuropsychologia.2017.11.033)
Supplement: Supplementary file 1 — Supplementary material [file mmc1.docx]

# Supplement

1. **List of Studies Included in the Meta-analyses**

Think/No-Think Task

| **First Author** | **Year** | **N** | **Journal** | **Preprocessing** | **Smoothing (mm)** |
| --- | --- | --- | --- | --- | --- |
| Anderson | 2004 | 24 | Science | realign, normalise | 6 |
| Benoit | 2012 | 18 | Neuron | realign, coregister, normalise | 8 |
| Benoit | 2012 | 18 | Neuron | realign, coregister, normalise | 8 |
| Benoit | 2015 | 16 | Journal of Cognitive Neuroscience | realign, coregister, normalise | 8 |
| Butler | 2010 | 14 | Cognitive, Affective, & Behavioral Neuroscience | realign, coregister, normalise | 6 |
| Depue | 2007 | 16 | Science | realign, normalise | 6 |
| Depue | 2015 | 21 | Cerebral Cortex | realign, normalise | 6 |
| Fawcett | In prep | 30 |  | realign, coregister, normalise | 8 |
| Gagnepain | 2014 | 24 | Proceedings of the National Academy of Sciences | realign, normalise | 10 |
| Gagnepain | 2017 | 24 | Journal of Neuroscience | realign, normalise | 10 |
| Levy | 2012 | 18 | Journal of Neuroscience | native space | 4 |
| Levy | In prep | 18 |  | realign, coregister, normalise | 8 |
| Liu | 2016 | 18 | Nature Communications | realign, coregister, normalise | 4 |
| Paz-Alonso | 2013 | 33 | Journal of Neuroscience | realign, normalise | 8 |
| Sacchet | 2016 | 16 | Cognitive, Affective, & Behavioral Neuroscience | realign, normalise | 4 |
| Schmitz | 2017 | 24 | Nature Communications | realign, coregister, normalise | 8 |

Stop-Signal Task

| **First Author** | **Year** | **N** | **Journal** | **Preprocessing** | **Smoothing (mm)** |
| --- | --- | --- | --- | --- | --- |
| Aron | 2006 | 13 | Journal of Neuroscience | realign, coregister, normalise | 5 |
| Aron | 2007 | 10 | Journal of Neuroscience | realign, coregister, normalise | 5 |
| Berkman | 2014 | 60 | Journal of Neuroscience | realign, coregister, normalise | 6 |
| Boecker | 2011 | 15 | Human Brain Mapping | realign, normalise | 8 |
| Boehler | 2010 | 15 | NeuroImage | realign, normalise | 8 |
| Cai | 2009 | 12 | Brain Research | realign, coregister, normalise | 8 |
| Cai | 2011 | 23 | PLos One | realign, coregister, normalise | 8 |
| Cai | 2014 | 19 | Human Brain Mapping | realign, coregister, normalise | 8 |
| Chamberlain | 2009 | 20 | Biological Psychiatry | realign, coregister, normalise | 8 |
| Chevrier | 2007 | 14 | Human Brain Mapping | native space | 6 |
| Chikazoe | 2009 | 22 | Journal of Neuroscience | realign, normalise | 8 |
| Cohen | 2010 | 9 | Frontiers in Human Neuroscience | native space | 5 |
| Congdon | 2014 | 62 | Psychiatry Research: Neuroimaging | realign, coregister, normalise | 5 |
| Cumins | 2011 | 50 | Molecular Psychiatry | realign, coregister, normalise | 9 |
| De Wit | 2012 | 37 | American Journal of Psychiatry | realign, coregister, normalise | 8 |
| Depue | 2015 | 21 | Cerebral Cortex | realign, normalise | 6 |
| Ghahremani | 2012 | 18 | Journal of Neuroscience | realign, coregister, normalise | 6 |
| Hendrick | 2010 | 60 | PLos One | realign, normalise | 10 |
| Hendrick | 2012 | 18 | Behaviour and Psychology | realign, normalise | 8 |
| Hughes | 2013 | 15 | Behavioural Brain Research | realign, coregister, normalise | 8 |
| Jahfari | 2011 | 20 | Journal of Neuroscience | realign, coregister, normalise |  |
| Lenartowicz | 2011 | 23 | Journal of Cognitive Neuroscience | realign, coregister, normalise | 5 |
| Leung | 2007 | 12 | Journal of Neuroscience | realign, coregister, normalise | 8 |
| Marco-Pallares | 2008 | 10 | Journal of Cognitive Neuroscience | realign, normalise | 8 |
| McNab | 2008 | 11 | Neuropsychologia | realign, coregister, normalise | 6 |
| Montojo | 2013 | 30 | Cerebral Cortex | realign, coregister, normalise | 5 |
| Passarotti | 2010 | 15 | Neuropsychologia | native space |  |
| Ramautar | 2006 | 16 | Brain Research | realign, normalise | 8 |
| Rubia | 2001 | 15 | NeuroImage | realign, normalise | 7.2 |
| Sagaspe | 2011 | 14 | NeuroImage | realign, normalise | 8 |
| Schel | 2014 | 14 | Frontiers in Human Neuroscience | realign, normalise | 8 |
| Sebastian | 2012 | 24 | Psychiatry Research: Neuroimaging | realign, coregister, normalise | 8 |
| Sebastian | 2013 | 49 | Neurobiology of Ageing | realign, coregister, normalise | 8 |
| Sebastian | 2013 | 24 | NeuroImage | realign, coregister, normalise | 8 |
| Sharp | 2010 | 26 | Proceedings of the National Academy of Sciences | realign, normalise | 5 |
| Tabu | 2012 | 13 | NeuroImage | realign, normalise | 6 |
| Van der Meer | 2011 | 19 | NeuroImage | realign, coregister, normalise | 10 |
| Xue | 2008 | 15 | Cerebral Cortex | realign, coregister, normalise | 5 |
| Zheng | 2008 | 20 | Journal of Cognitive Neuroscience | realign, coregister, normalise | 9 |

Go/No-Go Task

| **First Author** | **Year** | **N** | **Journal** | **Preprocessing** | **Smoothing (mm)** |
| --- | --- | --- | --- | --- | --- |
| Altshuler | 2005 | 13 | Biological Psychiatry | realign, normalise | 6 |
| Asahi | 2004 | 17 | European Archives of Psychiatry and Clinical Neuroscience | realign, coregister, normalise | 8 |
| Braver | 2001 | 14 | Cerebral Cortex | realign, normalise | 6 |
| Falconer | 2008 | 23 | Journal of Psychiatry & Neuroscience | realign, normalise | 8 |
| Fassbener | 2004 | 18 | Cognitive Brain Research | native space | 3 |
| Garavan | 1999 | 14 | Proceedings of the National Academy of Sciences | native space | 4.2 |
| Garavan | 2002 | 14 | NeuroImage | native space | 4.2 |
| Garavan | 2003 | 16 | NeuroImage | native space | 4.2 |
| Hester | 2004 | 15 | Journal of Cognitive Neuroscience | native space | 3 |
| Horn | 2003 | 19 | Neuropsychologia | realign, normalise | 8 |
| Kaladjian | 2007 | 21 | Schizophrenia Research | realign, normalise | 9 |
| Kaladjian | 2009 | 10 | Bipolar Disorder | realign, normalise | 9 |
| Kaladjian | 2009 | 20 | Psychiatry Research: NeuroImage | realign, normalise | 9 |
| Kelly | 2004 | 15 | European Journal of Neuroscience | native space | 3 |
| Kiehl | 2000 | 14 | Psychophysiology | realign, normalise | 8 |
| Konishi | 1998 | 5 | European Journal of Neuroscience | realign |  |
| Langenecker | 2007 | 22 | Biological Psychiatry | realign, coregister, normalise |  |
| Liddle | 2001 | 16 | Human Brain Mapping | realign, normalise | 8 |
| Maltby | 2005 | 14 | NeuroImage | realign, normalise | 12 |
| Mazzola-Pomietto | 2009 | 16 | Journal of Psychiatric Research | realign, coregister, normalise | 9 |
| McNab | 2008 | 11 | Neuropsychologia | realign, coregister, normalise | 6 |
| Mostofsky | 2003 | 48 | Cognitive Brain Research | realign, normalise | 7 |
| Mostofsky | 2003 | 28 | Cognitive Brain Research | realign, normalise | 7 |
| Roth | 2007 | 14 | Biological Psychiatry | realign, normalise | 10 |
| Rubia | 2001 | 15 | NeuroImage | realign, normalise | 7.2 |
| Rubia | 2006 | 23 | Human Brain Mapping | realign, normalise | 7.2 |
| Sebastian | 2012 | 24 | Psychiatry Research: NeuroImage | realign, coregister, normalise | 8 |
| Simoes-Franklin | 2010 | 16 | Human Brain Mapping | native space | 3 |
| Watanabe | 2002 | 11 | NeuroImage | realign, normalise | 12 |
| Zheng | 2008 | 20 | Journal of Cognitive Neuroscience | realign, coregister, normalise | 9 |
